# Links of Perceived Pornography Realism with Sexual Aggression via Sexual Scripts, Sexual Behavior, and Acceptance of Sexual Coercion: A Study with German University Students

**DOI:** 10.3390/ijerph19010063

**Published:** 2021-12-22

**Authors:** Barbara Krahé, Paulina Tomaszewska, Isabell Schuster

**Affiliations:** 1 Department of Psychology, University of Potsdam, 14476 Potsdam, Germany; paulina.tomaszewska@uni-potsdam.de; 2 Department of Education and Psychology, Free University of Berlin, 14195 Berlin, Germany; isabell.schuster@fu-berlin.de

**Keywords:** pornography use, realism, sexual aggression, sexual victimization, sexual scripts

## Abstract

Exposure to pornographic material has been linked to sexual aggression perpetration and victimization in a large body of research. Based on social learning theory and _3_A theory of script learning, this study contributes to this research by testing the hypothesis that the more realistic pornography is perceived to be by young adults, the more likely they are to experience and engage in sexual aggression. Two underlying pathways were proposed: one path via scripts and patterns of sexual behavior regarding consensual sexual interactions that contain established risk factors for sexual aggression victimization and perpetration, and a second path via the acceptance of sexual coercion. In a cross-sectional study, 1181 university students in Germany (762 female; 419 male) completed measures of pornography use and perception, risky sexual scripts and sexual behavior, and acceptance of sexual coercion. As predicted, pornography realism was a positive predictor of risky sexual scripts, risky sexual behavior, and acceptance of sexual coercion. Indirect links with sexual aggression victimization and perpetration were found via both pathways. No gender differences in the associations were found. The implications for media literacy interventions addressing the realism of pornography are discussed.

## 1. Introduction

Pornography use is ubiquitous among adolescents and young adults around the world, especially since it has become easily accessible via the internet (e.g., [1,2,3]) and in video games [4]. Pornography may be defined as sexually explicit material intended to arouse [5]. Prevalence studies indicate that up to 90% of adolescents and young adults have been voluntarily exposed to pornographic media contents. A recent review of the prevalence and frequency of young men’s pornography use revealed weekly user rates of up to 80% [6]. There is consistent evidence that regular pornography use is more common among men than among women, but women’s user rates are still substantial [7,8].

A key motivation for pornography use is the expectation to acquire relevant information about sexuality and sexual relationships from these sources, identifying pornography as a learning tool, especially for adolescents and young adults [9,10]. As with any learning tool, what is learned depends critically on the content and the way in which it is presented [11]. Content analyses of pornographic material have revealed several characteristics that may be considered problematic in terms of being associated with negative outcome variables. For example, the content analysis of 50 videos topping the list of pornography sales and rentals in 2004 and 2005 [12] established that 88% of all coded scenes showed acts of physical aggression, such as spanking or gagging, and 48% contained verbal aggression. Most of the aggressive behaviors were shown by a male actor towards a female target, and 95% of the targets responded either with expressions of pleasure or in a neutral way to the aggressive treatment. Another content analysis examined reactions shown by adolescent and adult female actors to sexually aggressive male behavior and found that 90% of the teenage performers and more than 80% of the adult performers reacted with pleasure [13]. Consistent with this finding, another content analysis revealed that the importance of obtaining consent is often undermined in pornographic depictions of sexual interactions [14].

In line with the proposition that pornographic material may be a source of observational learning, a large body of evidence has pointed to an association between pornography use and sexual behavior, sexual aggression as well as attitudes condoning sexual aggression. The current study investigated the associations between exposure to pornography and sexual aggression perpetration and victimization in a large sample of university students in Germany. Beyond examining a direct link, we focus on two potential mechanisms by which pornography realism may be linked to sexual aggression perpetration and victimization. The first mechanism is proposed to operate via sexual scripts and sexual behavior defined as risky in terms of established predictors of sexual aggression perpetration and victimization, and the second is proposed to operate via the acceptance of sexual coercion. Sexual aggression is defined as behavior carried out with the intent or result of making another person engage in sexual activity despite his or her unwillingness to do so [15]. Rather than focusing on the frequency of pornography use, we considered the pornography realism, measured by a combination of frequency of use and perception as realistic, as the critical variable from a social-learning point of view.

### 1.1. Pornography Use, Perceived Realism and Sexual Aggression

A large body of evidence using different methodologies suggests a positive association between the frequency of pornography use and sexual aggression perpetration (see 7 for a review). A meta-analysis including 22 correlational and longitudinal studies from seven countries that linked pornography use to sexual aggression perpetration in general-population samples found a significant effect size of *r* = 0.28 [16]. No significant differences were found in relation to age, gender, country, or design (correlational vs. longitudinal). In addition, significant longitudinal associations of frequency of pornography use with sexual victimization were found in different countries (e.g., [17,18,19]). Although support for a direct link between pornography use and sexual aggression perpetration has been questioned by a recent meta-analysis [20] and longitudinal studies from Croatia [21,22], other longitudinal studies have shown pornography use to be a moderator magnifying the link with other risk factors [23], or showing indirect associations via sexuality-related cognitions or behaviors [19].

From the perspective of social learning theory, the transfer from contents observed in the media to users’ own behaviors should be more likely the more they perceive the observed contents to be realistic [24]. Moreover, the more often pornographic content is observed, the more realistic it should appear [25]. Therefore, perceived realism of pornographic depictions of sexuality should be an important condition for the path from pornography exposure to sexuality-related cognitions and attitudes, sexual behavior, and sexual aggression perpetration and victimization. In line with this reasoning, a study with heterosexual German adults found that pornography use predicted condom-less sex only among participants who saw pornography as a source of information [26]. A three-wave longitudinal study with Dutch adolescents found that pornography use predicted sexually permissive attitudes only in participants scoring high on a measure of perceived realism of pornographic depictions of sex [27]. A further study found positive associations between the perceived realism of pornography and the odds of perpetrating sexual aggression for both male and female participants [28]. In combination, this evidence is consistent with social learning theory in that attitudes and behavior are more likely to be acquired the more realistic they are perceived to be. It is also in line with the proposition that pornographic material “normalizes” the depicted forms of sexual relationship and sexual behavior, which facilitates their inclusion into viewers’ sexual scripts [29].

### 1.2. Pornography Use, Risky Sexual Scripts, and Risky Sexual Behavior

Rather than imitating specific behaviors, pornography provides a basis for developing more complex and comprehensive cognitive representations of sexual interactions, as captured in the construct of “sexual scripts” [30]. Sexual scripts contain descriptive features and normative elements of sexual interactions rooted in the predominant social constructions of sexuality in a given culture. They can be specific to certain types of sexual interactions, such as the “hook-up script” or the “rape script” [31,32], and they function as guidelines for sexual behavior.

A framework for integrating the different strands of pornography research in relation to sexual scripts is offered by Wright’s _3_A theory, standing for *acquisition*, *activation*, and *application* [33,34]. Voluntary exposure to pornography is seen as shaping individuals’ sexual scripts, which are activated in sexual situations to interpret the situation. The activated script is evaluated as to its suitability for enactment in the situation. If the evaluation is positive, the script will be applied to the planning and performance of sexual behavior.

We refer to sexual script as “risky” if they include features that have been identified consistently in the scientific literature as increasing the probability of sexual aggression victimization and perpetration. Specifically, we consider three such factors as elements of risky sexual scripts: alcohol use during sexual encounters, sex with casual partners, and ambiguous communication of sexual intentions [35,36,37]. As shown by content analyses, these features are often presented in pornographic media [38,39]. Accordingly, studies found significant associations between pornography use and sexual scripts, defined as risky with respect to increasing the odds of sexual aggression victimization and perpetration [17,19,40,41]. These studies also yielded significant paths from risky sexual scripts to risky sexual behavior, supporting the conceptualization of scripts as guidelines for behavior. Other studies showed direct links between exposure to pornography and corresponding patterns of sexual behaviors [42,43,44,45,46].

### 1.3. Pornography Use and Acceptance of Sexual Coercion

As shown by the content analyses discussed above, many pornographic media contain violence against women that is seemingly enjoyed by the targets or show initially reluctant women changing their mind after forceful attempts by the man to engage in sexual contact. Such presentations may foster the view that women enjoy aggressive sexual tactics and that they are usually willing to have sex even if they initially reject a man’s advances. Supporting this line of reasoning, a review found evidence that both sexually explicit material in general, and material depicting rape or physical harm in a sexual context in particular, were linked to attitudes condoning sexual violence and physical dating violence [47]. Recent meta-analytic reviews found significant links between exposure to pornography, especially violent pornography (defined as depictions of intentional attempts by individuals to inflict extreme physical harm on others, such as rape), and rape myth acceptance and normative beliefs condoning violence [48,49]. Moreover, greater acceptance of sexual aggression and rape myth acceptance have been linked to higher odds of both sexual aggression perpetration and victimization, as shown in recent reviews [50,51]. Moreover, the normative acceptance of sexual coercion has also been linked to sexual victimization [52].

### 1.4. The Role of Gender

A consistent body of evidence found that the use of pornographic material is more common among men than women from adolescence onwards [7,53]. However, despite this difference in mean levels, perceived realism does not seem to differ between gender groups [27,54]. Some gender-specific findings were found regarding the associations of pornography use with sexuality-related attitudes and sexual behavior [16,28,54,55]. Moreover, evidence of a link between exposure to sexually explicit material and risky sexual behavior in terms of condom-less sex was found for sexual minority men [56,57]. Therefore, we proposed that the associations between pornography use, sexual scripts and behavior, acceptance of sexual coercion, and sexual aggression perpetration and victimization would be found for both men and women.

### 1.5. The Current Study

Based on the theorizing and findings summarized above, the current study was designed to examine the associations between pornography realism, defined and operationalized by the product of perceived realism and frequency of use, and sexual aggression perpetration and victimization in a large sample of university students in Germany. To achieve this objective, two pathways were distinguished by which pornography realism may affect sexual aggression perpetration and victimization. The first pathway was assumed to operate via shaping risky scripts for consensual sex, defined by features linked to an increased risk of sexual aggression perpetration and vulnerability to victimization. A separate path was assumed to operate via shaping attitudes accepting sexual coercion. Going beyond previous evidence, we collected both perpetration and victimization data from men and women to test the following hypotheses:

**Hypothesis** **1.** *The higher pornography realism, the more likely participants are to report sexual aggression perpetration and victimization.*

**Hypothesis** **2.** *The higher pornography realism, the riskier participants’ sexual scripts are for consensual sex and the greater their acceptance of sexual coercion.*

**Hypothesis** **3.** *The riskier participants’ sexual scripts are for consensual sex, the riskier their sexual behavior in consensual sexual interactions.*

**Hypothesis** **4.** *The riskier sexual behavior participants show in consensual sexual interactions, the more likely they are to perpetrate and experience sexual aggression.*

**Hypothesis** **5.** *The more accepting participants are of sexual coercion, the more likely they are to perpetrate and experience sexual aggression.*

**Hypothesis** **6.** *Higher pornography realism is indirectly linked to higher odds of perpetrating and experiencing sexual aggression via two underlying mechanisms. The first is via risky sexual scripts and risky sexual behavior, the second via the acceptance of sexual coercion.*

Based on past findings, we proposed that the associations between pornography realism, risky sexual scripts and behavior, acceptance of sexual coercion, and sexual aggression perpetration and victimization would be found for both men and women. 

## 2. Method

### 2.1. Sample

The study was advertised as a study on young adults’ competence in sexual situations. Invitations to participate in the study were sent out to students enrolled in four higher education institutions in the Brandenburg and Berlin region in Germany through the respective student offices or student associations. Students interested in participating registered in a data bank created for the purposes of this study and were sent the link to the online survey upon registration. A total of 1181 (762 female, 419 male) university students provided data for this study. This number excluded seven respondents who indicated their sex as “other” because the number was too small to warrant separate analysis. Participants were randomly assigned to either an intervention condition to reduce risk factors and rates of sexual aggression perpetration and victimization or a no-intervention control condition. All participants were included in the current analysis because the data were collected at baseline, prior to the presentation of any intervention material and without participants’ awareness about their condition membership. The mean age of the sample was 22.6 years (*SD* = 3.52; range: 18–35 years). Almost all participants (92.9%) were German nationals.

In terms of sexual and relationship experience, 89% of the sample had coital experience, 2.5% did not, the remaining 7.5% did not answer this question. The mean age at first sexual intercourse was 16.8 years (*SD* = 2.20). The mean number of casual sex partners was 6.49 (*SD* = 10.50), the mean number of sex partners in a steady relationship was 2.44 (*SD* = 1.87). Most participants (87%) were or had been in a steady relationship experience at the time of the survey and/or in the past. The majority of participants (78.6%) described their sexual orientation as heterosexual, 5.7% as homosexual, 11.1% as bisexual, and 4.7% did not answer the question. Most reported exclusively heterosexual contacts (67.0% of women, 68.9% of men), 1.6% of women and 6.6% of men reported exclusively same-sex contacts, and 26.3% of women and 16.3% of men reported both heterosexual and same-sex contacts, 5.1% of women and 8.3% of men reported neither opposite-sex nor same-sex contact. The mean age at first intentional exposure to pornography was 15.1 (*SD* = 2.97), with women being older (*M* = 15.9, *SD* = 3.20) than men (*M* = 13.9, *SD* = 2.07), *t* (988) = 10.89, *p* < 0.001.

### 2.2. Instruments

#### 2.2.1. Frequency of Use and Perception of Pornography as Realistic

Frequency of pornography use was measured by the following item: “Have you ever deliberately watched media with explicit sexual content (i.e., images, videos, or films of sexual acts, such as sexual intercourse, oral sex, masturbation etc.)?” Responses were made on a five-point scale ranging from 1 (*never*) to 5 (*very often*). Perception of pornography was measured by three items based on past research [25]: “The way sexuality is presented in pornographic media is quite realistic.”; “By watching sexual images and videos, one learns how to behave in sexual situations.”; and “Pornographic media convey valuable information about sex.” Responses were made on a five-point scale ranging from 1 (*do not agree at all*) to 5 (*completely agree*). Responses were averaged across the three items to yield a total score of perceived realism of pornography, α = 0.76. 

To create an overall score of pornography realism, we multiplied the perception of pornography score, aggregated across the three items, by the reported frequency of pornography use. As both measures had a response scale ranging from 1 to 5, the resulting multiplicative score had a range from 1 to 25.

#### 2.2.2. Risky Sexual Scripts 

To arrive at a measure of risky sexual scripts for consensual sexual encounters, a two-part measure was used, following past research [36,41]. The first part measured the descriptive content of risky scripts with regard to the following scenario: “You spend the evening together with a man/woman. In the course of the evening, you sleep together for the first time.” Participants received a tailored version depending on their sexual experience background: Women with exclusively heterosexual experiences and men with exclusively same-sex experiences received the version referring to a male partner, men with exclusively heterosexual experiences and women with exclusively same-sex experiences received the version referring to a female partner, participants with both opposite- and same-sex experiences received a gender-neutral version referring to “a person”. All participants were instructed to imagine themselves in this situation and indicate how likely a total of 10 features would be present in such a situation in general (i.e., generalizing across their personal experiences): (a) casual sex (3 items, example item: “How likely is it that you would have been on a date with the man/woman prior to that evening?”), reverse coded, (b) alcohol use (4 items, example item: ”How likely is it that you would have drunk alcohol in that situation?”), and (c) ambiguous communication of sexual intentions (3 items, example item: “How likely is it that you would explicitly ask the man/woman whether or not he/she wants to sleep with you?”; reverse coded). Responses were made on a five-point scale ranging from 1 (*very unlikely*) to 5 (*very likely*) and were averaged across the 10 items to yield an overall score of descriptive script elements, α = 0.68.

The second component of the script measure addressed the normative endorsement of the script elements with five items referring to the same three categories of (a) casual sex, (2 items, example item: “I find it OK to have sex with a man/woman without having been on a date with him/her before.”), (b) alcohol use (2 items, example item: “When I have sex with a man/woman, I don’t mind if he/she has had too much to drink.”), and (c) ambiguous communication of sexual intentions (1 item: “For me, it is clear that you talk with your partner to agree about sleeping together”, reverse coded). Responses to the normative items were made on a five-point scale ranging from 1 (*do not agree at all*) to 5 (*completely agree*). To create an overall score of the normative evaluation of script elements, responses were averaged across the five items (α = 0.67). A final score reflecting risky sexual scripts was calculated for each participant by multiplying the mean of the descriptive script items by the mean of the normative script items, following previous research [36,41]. The resulting score had a range from 1 to 25. 

#### 2.2.3. Risky Sexual Behavior

To measure the extent to which the elements of the sexual scripts for consensual scripts were reflected in actual sexual behavior, nine items were used, derived from previous research [35,58]. Participants were asked to indicate how often they had shown a particular behavior when they had sex in the past, using a five-point scale from 1 (*never*) to 5 (*very often*). Three items referred to casual sex (example item: “How often have you had sex with a man/woman on your first date?”), four items referred to alcohol use in sexual encounters (example item: “How often did you have too much to drink in situations in which you had sex?”), and two items referred to the ambiguous communication of sexual intentions (example item: “How often have you first said ’no’ to a sexual encounter even though you actually wanted it?”). Responses were averaged across the nine items to yield a total risky behavior score. As risky sexual behavior is an additive score, calculating internal consistency is not meaningful for this measure.

#### 2.2.4. Acceptance of Sexual Coercion

The extent to which participants found the use of sexual coercion acceptable was measured with seven items derived from previous research [36,41]. Participants were presented with the following scenario, tailored to their gender and sexual experience by referring to their own perspective in interaction with a partner of the opposite or the same sex: “Imagine Alexander/Hannah wants to have sex with Hannah/Alexander, but she/he clearly and unequivocally says “no”. Under what circumstances would you find it okay for Alexander/Hannah to get Hannah/Alexander to sleep with him/her nonetheless?”. The seven items presented justifications for one person’s persistence despite the other person’s “no” (example items: “If Hannah is drunk or stoned; “If Hannah slept with Alexander before”). Responses were made on a five-point scale ranging from 1 (*under no circumstances*) to 5 (*definitely*). Responses were averaged across the seven items to yield a total score, α = 0.87.

#### 2.2.5. Sexual Aggression Victimization and Perpetration

Sexual aggression victimization and perpetration were measured with the Sexual Aggression and Victimization Scale (SAV-S) [59]. Building on the Sexual Experiences Survey (SES) [60], the SAV-S uses behaviorally specific items to elicit victimization and perpetration reports for three coercive strategies (use or threat of physical force, exploitation of the other person’s inability to resist, for example due to alcohol use, and verbal pressure) and four sexual acts (sexual touch, attempted penetration, completed penetration, other sexual acts, for example oral sex). Going beyond the SES, questions about each combination of coercive strategy and sexual act are asked for three relationship constellations (former/current partner, friend/acquaintance, stranger). The combination of three coercive strategies, four sexual acts, and three relationship constellation results in a total of 36 victimization and 36 parallel perpetration items. For each item, participants indicated whether they had experienced (victimization) or engaged in (perpetration) that particular behavior since their 14th birthday, the age of consent in Germany. Response options were 0 (*no*) and 1 (*yes*). The format of the SES is illustrated in the Appendix A. The SAV-S was validated in several studies in different countries [61,62,63,64]. As with the script measure, item wordings were matched to participants’ sexual experience background: Women with exclusively heterosexual experiences and men with exclusively same-sex experiences received the version referring to a male partner, men with exclusively heterosexual experiences and women with exclusively same-sex experiences received the version referring to a female partner, participants with both heterosexual and same-sex experiences received a gender-neutral version referring to “a person”.

To arrive at a measure of sexual aggression victimization, we first categorized participants into nonvictims (“no” responses to all victimization items) and victims (“yes” response to at least one victimization item). To take differences in the severity of the victimization experience into account, an additional five-level score was created in which each participant was classified in terms of the most serious reported experience of victimization, following a procedure used in previous research with the SAV-S (e.g., [59,63]) and an analogous approach used for the Sexual Experiences Survey [60,65]. The categories were defined as follows: (0) *No victimization* (“no” responses to all SAV-S items); (1) *Sexual contact* without penetration (i.e., sexual touch) or other sexual acts, but no sexual coercion, attempted rape, and rape; (2) *Sexual coercion*, i.e., attempted or completed vaginal or anal penetration or other sexual acts using verbal pressure, but no attempted or completed rape; (3) *Attempted rape*, i.e., attempted vaginal, or anal penetration through exploitation of the victim’s inability to resist or threat or use of physical force, but no completed rape; and (4) *Completed rape*, i.e., completed vaginal or anal penetration through exploitation of the victim’s inability to resist or threat or use of physical force. 

For sexual aggression perpetration, the frequencies for the five categories (except for the no-perpetration category) were too low to warrant a graded severity score. Therefore, a dichotomous score was created in the same way as for the dichotomous victimization score (*nonperpetrators*: “no” responses to all perpetration items; *perpetrators*: “yes” response to at least one perpetration item).

### 2.3. Procedure and Plan of Analysis

The study was conducted according to the guidelines of the Declaration of Helsinki and approved by the Ethics Committee of the first author’s university. Participants were informed that they could terminate the study at any point. They had to give active consent on the first page of the survey before being able to proceed to the questions. On each page of the SAV-S, a “Help” button was placed that led to a list of support agencies for victims and perpetrators of sexual aggression. Participants received an online shopping voucher in return for their participation. The broader intervention study from which the current data were taken was pre-registered as an “as predicted” study on the website of the Open Science Framework, https://osf.io/cg6xq (accessed on 15 December 2021).

To test the proposed associations of pornography realism with risky sexual scripts, risky sexual behavior, acceptance of sexual coercion and sexual aggression perpetration and victimization, paths models were examined with the Mplus software, version 8.6 (Muthén & Muthén, Los Angeles, CA, USA) [66]). As the outcome variables of perpetration (nominal) and victimization (ordinal) were categorical, we used the wlsmv estimator in the analyses. Indirect paths were tested via 10,000 bias-corrected bootstrapped standardized confidence intervals.

## 3. Results

### 3.1. Descriptive Results and Correlations

Means and standard deviations for all study variables in the total sample and the two gender groups are presented in Table 1. Men scored significantly higher than women on the measure of pornography realism, held riskier sexual scripts for consensual sex, and had a higher prevalence of sexual aggression perpetration. The percentage of victimization and the mean severity score were significantly higher for women than for men. No gender differences were found for risky sexual behavior and acceptance of sexual coercion.

The bivariate correlations between the predictor variables for women and men are shown in Table 2. Pornography realism showed significant positive correlations with risky sexual scripts, risky sexual behavior, and acceptance of sexual coercion in both gender groups. Risky sexual scripts and risky sexual behavior were significantly correlated with each other in both gender groups and with the acceptance of sexual coercion in men. Except for the correlation between pornography realism and acceptance of sexual coercion, all bivariate correlations were significantly higher for men than for women.

### 3.2. Path Analyses

**Figure 1:**
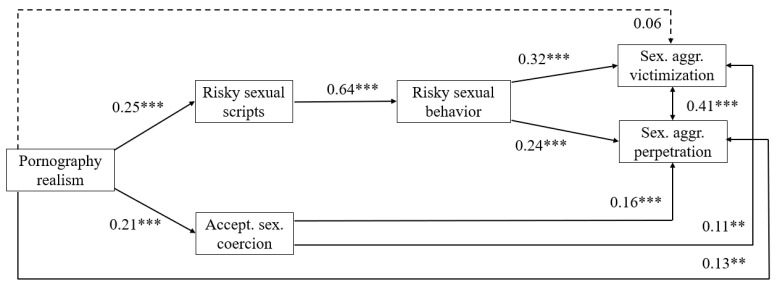
Path model linking pornography realism to sexual aggression (standardized coefficients). *** *p* < 0.001; ** *p* < 0.01 (two-tailed). Model fit: *Chi*^2^ (*df* = 5) = 37.33, *p* < 0.001; CFI = 0.985, RMSEA = 0.042 (90% C.I. 0.019; 0.067), SRMR = 0.016. All paths controlled for gender.

To test the proposed path model, we first estimated a multi-group model by gender in which the paths were allowed to vary between men and women. As 17 participants had missing values on all variables included in the model and 10 participants had missing values on the exogenous predictor variables, they were excluded from the model estimation, yielding a sample size of 1154 for this analysis. The multi-group model showed a good fit with the data, *Chi*^2^ (*df* = 10) = 37.33, *p* < 0.001; CFI = 0.957, RMSEA = 0.069 (90% C.I. 0.046; 0.093), SRMR = 0.030. Paths were tested for significant differences via the Difftest option, which showed that none of the paths differed significantly between men and women. Therefore, to account for gender differences in the means of pornography realism and risky sexual scripts, we estimated a single-group model in which all paths were controlled for gender. This model showed a very good fit, *Chi*^2^ (*df* = 5) = 15.29, *p* < 0.01; CFI = 0.985, RMSEA = 0.042 (90% C.I. 0.019; 0.067), SRMR = 0.016, and was accepted as the final model, presented in Figure 1.

The direct link between pornography realism was significant with sexual aggression perpetration, but not with sexual aggression victimization, partly supporting Hypothesis 1. The two proposed pathways were found to be significant. As predicted in Hypothesis 2, positive paths were found from pornography realism to risky sexual scripts for consensual sex and acceptance of sexual coercion. Consistent with Hypothesis 3, risky scripts for consensual sex significantly predicted risky sexual behavior. The predicted positive paths from risky sexual behavior to sexual aggression perpetration and victimization (Hypothesis 4) were also confirmed, as were the paths from the acceptance of sexual coercion to perpetration and victimization (Hypothesis 5). Finally, as predicted in Hypothesis 6, pornography realism indirectly predicted perpetration and victimization, both via risky sexual scripts and risky sexual behavior and via the acceptance of sexual coercion. The indirect paths are shown in Table 3.

## 4. Discussion

Although users of pornography generally perceive positive effects of pornography consumption [67], empirical studies have yielded evidence of a range of negative outcomes in the domain of sexuality and aggression. The current study analyzed the associations between pornography realism and sexual aggression perpetration and victimization, mediated by two distinct pathways: via risky sexual scripts and risky sexual behavior, and via the acceptance of sexual coercion. Using social learning theory and the _3_A theory of script learning [24,33] as a conceptual basis, we predicted that exposure to pornography would be a source of observational learning, reflected in scripts as cognitive representations of sexual interactions, sexual behavior, and attitudes condoning sexual coercion. Additionally, referring to social learning theory, we proposed that learning from pornography would be more likely the more realistic the pornographic contents were perceived to be. Therefore, we used a multiplicative index of frequency of use and perception of pornography as our measure of pornography realism. Self-reported sexual aggression perpetration and victimization, both assessed in male and female participants, were the criterion variables. 

The first hypothesis, predicting a direct association between pornography realism and sexual aggression, was confirmed for perpetration, but not for victimization. This is a novel finding because we were unable to find earlier studies linking pornography to perpetration and victimization reports obtained from the same individuals. A tentative explanation could be that observational learning is more likely to focus on the actor perspective than the target perspective. Moreover, the shared variance between perpetration and victimization, reflected in a moderate positive correlation, may have rendered the weaker link of pornography realism with victimization nonsignificant. Further research is needed to replicate these differential associations. However, the lack of a direct association between pornography realism and sexual victimization is qualified by significant indirect associations in line with our theoretical reasoning, as discussed below. The meta-analytic conclusion by Ferguson et al. [20] that evidence for a link between pornography use and sexual aggression is weak needs to be evaluated including evidence of indirect effect via sexual scripts, sexual behavior and sexuality-related attitudes referring to consensual sexual interactions.

Hypothesis 2, predicting that higher pornography realism would show a significant positive association with both risky sexual scripts and acceptance of sexual coercion was supported by the data, in line with findings from previous research conducted in different countries [41,42]. The finding is also consistent with social learning theory and sexual script theory, proposing that observational learning and vicarious reinforcement may shape cognitive representation of sexual interactions as well as sexual behavior. It is worth noting that the conceptualization of a link between pornography use and sexual scripts does not assume a one-to-one copying of sexual practices into viewers’ behavioral repertoire, but rather a more general association with the cognitive representation of sexual interactions. This was shown, for instance, in a recent meta-analysis that found that pornographic material that did not contain condom-less sex was related to engaging in condom-less sex, whereas material that contained condom-less sex was unrelated to participants’ engagement in sex without condoms [68].

Supporting Hypothesis 3, riskier sexual scripts were associated with riskier sexual behavior. Furthermore, risky sexual behavior was positively associated with both sexual aggression victimization and perpetration, supporting Hypothesis 4. This pattern of findings corroborates previous evidence from different countries [35,69,70] and underlines the conceptualization of sexual scripts as guidelines for sexual behavior.

Additionally, consistent with our prediction in Hypothesis 5 and with previous studies, greater acceptance of sexual coercion was positively associated with both outcomes [71,72]. Individuals who consider sexual aggression justified under certain circumstances may be more likely to use aggressive means to obtain sexual contacts, and their greater tolerance of coercive strategies for obtaining sex may also make it more likely for them to experience sexual interactions involving the threat of coercion.

Finally, as predicted in Hypothesis 6, higher pornography realism indirectly predicted sexual aggression perpetration and victimization via the sequence of risky sexual scripts and risky sexual behavior, and via the acceptance of sexual coercion, as shown in previous longitudinal studies [17,19]. These patterns of association are in line with the proposed role of pornographic media contents in shaping cognitive, behavioral, and attitudinal constructs related to sexual aggression perpetration and victimization. Whether the proposed sequence of these constructs implies a causal role in explaining perpetration and victimization cannot be answered on the basis of the current cross-sectional data, but longitudinal evidence from other sources points in this direction [17,40]. Despite significantly higher scores for men on the measure of pornography realism compared to women, no significant gender differences were found in the proposed path model, suggesting that the underlying processes linking pornography to sexual aggression perpetration and victimization operate in similar ways in men and women. This finding has implications for designing intervention measures, suggesting that both men and women may benefit from strategies for challenging the perception that pornographic media portray a realistic picture of sexual interactions. 

### Limitations and Implications

Although the findings support most of our predictions, some limitations need to be mentioned about the study. First, the data are based on a convenience sample of university students from several state-funded universities. There is no reason to assume that the student body of these universities would be nonrepresentative of the student population in Germany, but self-selection into the study cannot be ruled out. If this had been the case, it would primarily affect the prevalence rates of perpetration and victimization, and to a lesser extent the associations between the study variables that were at the core of the analyses. Nonetheless, the findings need further corroboration in independent samples. A second limitation is the cross-sectional nature of the data, which rules out conclusions about causal or temporal pathways. Third, the internal consistencies for the two parts of the script measure were just below the accepted threshold of 0.70. Furthermore, we used a broad question to assess frequency of pornography use, which did not distinguish between different types of pornographic content, such as violent vs. nonviolent or mainstream vs. feminist pornography [11,48]. A more fine-grained measure of the type of pornography used would enable future research to examine differential associations with sexual aggression perpetration and victimization [7]. Recent methodological critiques of the field of pornography research have called for greater efforts to create validated measures, which are needed to facilitate a better comparability of findings across future studies [73,74].

Despite these limitations, the consistency of the current findings with evidence from longitudinal studies points to the potential efficacy of interventions designed to change perceptions of pornography as realistic as well as sexual scripts for consensual sex to reduce the odds of sexual aggression perpetration and victimization. Existing programs to enhance porn literacy have found promising results [75,76].

## 5. Conclusions

In view of the widespread use of pornography among adolescents and young adults across the world, the associations detected in the present study with risky sexual scripts and behavior, acceptance of sexual coercion, and sexual aggression and victimization join a large body of international research on problematic aspects of pornography use. These findings call for interventions seeking to break the link between pornography use and sexual aggression and its mediating variables and also reduce other potentially harmful consequences, such as sexual objectification, sexual dissatisfaction, and unsafe sexual practices [77]. Porn literacy interventions have been developed and evaluated in recent studies [75,76]. Based on a social-learning perspective, interventions should enable users to develop critical thinking skills and challenge the perception that pornographic contents portray a realistic and normative image of sexual relations.

## Figures and Tables

**Table 1:** Means and standard deviations of all model variables.

Variable (Range)	Total Sample *M* (*SD*)	Men *M* (*SD*)	Women *M* (*SD*)
Pornography realism ^+^ (1–25)	5.45 (3.43)	7.59 (3.68) ^a^	4.26 (2.61) ^b^
Risky sexual scripts ^++^ (1–25)	7.11 (3.01)	7.43 (3.11) ^a^	6.94 (2.95) ^b^
Risky sexual behavior (1–5)	2.04 (0.60)	2.02 (0.62)	2.05 (0.59)
Acceptance of sexual coercion (1–5)	1.42 (0.58)	1.42 (0.60)	1.42 (0.57)
Sexual victimization (0–4)	1.16 (1.41)	0.76 (1.22) ^a^	1.38 (1.46) ^b^
Sexual aggression victimization (% yes)	53.4	37.7 ^a^	62.1 ^b^
Sexual aggression perpetration (% yes)	12.3	17.7 ^a^	9.4 ^b^

Note. Critical *p* for comparisons of means between gender groups: 0.05/4 = 0.0125. ^a,b^ Values are significantly different between men and women. ^+^ Multiplicative score: frequency of use (1–5) × perception of pornography (1–5). ^++^ Multiplicative score: descriptive elements (1–5) × normative elements (1–5).

**Table 2:** Bivariate correlations between the predictor variables.

Construct	1	2	3	4
1. Pornography realism	-	**0.29** ***	**0.23** ***	0.22 ***
2. Risky sexual scripts	**0.15** ***	-	**0.59** ***	**0.25** ***
3. Risky sexual behavior	**0.10** *	**0.67** ***	-	**0.17** **
4. Acceptance of sexual coercion	0.15 ***	**0.02**	**0.01**	-

Note. Correlations for men above the diagonal, correlations for women below the diagonal. *** *p* < 0.001; ** *p* < 0.01, * *p* ≤ 0.01 (two-tailed). Correlations in bold are significantly different between men and women.

**Table 3:** Significant indirect paths in the model based on standardized bias-corrected bootstrapped confidence intervals.

Indirect Paths	ß	99% C.I.
Pornography realism -> Risky script -> Risky behavior -> *Victimization*	0.050	0.020; 0.076
Pornography realism -> Risky script -> Risky behavior -> *Perpetration*	0.038	0.019; 0.067
Pornography realism -> Acceptance of sexual coercion -> *Victimization*	0.022	0.005; 0.046
Pornography realism -> Acceptance of sexual coercion -> *Perpetration*	0.033	0.010; 0.066

## Data Availability

The data presented in this study are available on request from the corresponding author. The data are not publicly available because the authors’ data analysis is still ongoing.

## Appendix A

Format of the SAV-S (Version for women with exclusively heterosexual and men with exclusively same-sex experiences).

### Appendix A.1. Victimization

1. Has a man ever made (or tried to make) you have sexual contact with him against your will **by threatening to use force or by harming** you (*e.g., by hurting you, holding you down or threatening to do so)*?
  (a) My **current or former partner** in a steady relationship to engage in…
    … sexual touch
    … attempted intercourse
    … completed intercourse
    … other sexual acts (e.g., oral sex)
  (b) A **friend** or **acquaintance** to engage in…
    … sexual touch
    … attempted intercourse
    … completed intercourse
    … other sexual acts (e.g., oral sex)
  (c) A **stranger** (e.g., someone I met at a club) to engage in…
    … sexual touch
    … attempted intercourse
    … completed intercourse
    … other sexual acts (e.g., oral sex)
    (*Yes* or *No* answers to each item)
2. Has a man ever made (or tried to make) you have sexual contact with him against your will **by exploiting the fact that you were unable to resist** (*e.g., after you had had too much alcohol or drugs*)?
  … + items (a), (b), (c) as above
3. Has a man ever made (or tried to make) you have sexual contact with him against your will **by putting verbal pressure on you** (*e.g., by threatening to end the relationship or spreading lies*)?
  … + items (a), (b), (c) as above

### Appendix A.2. Perpetration: Same Items from the Actor Perspective

Have you ever made (or tried to make) a man have sexual contact with you against his will **by threatening to use force or by harming him** (*e.g., by hurting him, holding him down or threatening to do so*)?

Parallel versions for women with exclusively same-sex contacts and men with exclusively heterosexual experiences referring to “a woman” instead of “a man”. Gender-neutral version referring to “a person” for participants with both opposite- and same-sex experiences.
